# Abnormal conductivity behavior in porous lead telluride films

**DOI:** 10.1186/1556-276X-7-442

**Published:** 2012-08-08

**Authors:** Sergey P Zimin, Egor S Gorlachev, Fedor O Skok

**Affiliations:** 1 Microelectronics Department, Yaroslavl State University, Sovetskaya Street 14, Yaroslavl, 150000, Russia; 2 Yaroslavl Branch of the Institute of Physics and Technology of Russian Academy of Sciences, Universitetskaya Street 21, Yaroslavl, 150007, Russia

**Keywords:** Porous semiconductors, Porosity, Lead telluride, Electrical conductivity, 81.05.Rm, 71.20.Nr, 72.60. + g

## Abstract

We report the experimental observation of the novel phenomenon of the resistivity decrease in porous PbTe layers during the pore formation process. Investigations were performed on the n-PbTe films with 2.3-μm thickness, which were near the point of the conductivity-type inversion at room temperature. Anodic electrochemical treatment for the porous layers with 41% to 52% porosity fabrication was performed using a KOH-based Norr electrolyte solution. For the porous lead telluride layers, the resistivity value at 300 K decreased 2.5 to 3 times. For the explanation of the observed phenomenon, a physical model is proposed which takes into account the Pb/Te ratio change during the anodic treatment.

## Background

One of the most urgent problems in the field of a new porous semiconductor material synthesis is the systematic study of the change of the electrical conductivity during the pore formation. In the classical cases, the processes of the pore formation result in the resistivity increase, which is due to the additional charge carrier scattering at the pores, the processes of the charge carrier depletion in the areas around the pores, quantum size effects, oxidation processes, etc. [1,2].

Recently, we have demonstrated that the formation of a porous lead telluride (PbTe) layer using anodic electrochemical treatment method is accompanied by the changes of the ratio between metal and chalcogen atoms [3]. This process is in strong degree determined by the anodic treatment conditions and by the initial material Pb/Te ratio. Secondary ion mass spectrometry investigations have shown that, in most cases, the tendency of the increase of the metal concentration with respect to chalcogen takes place. It is well known that, in lead chalcogenides, the concentration of the charge carriers is defined by a deviation from stoichiometry, with the abundance of lead resulting in the increase of the concentration of electrons. Under these conditions, theoretically, there are possibilities of an abnormal conductivity behavior when porous lead telluride would demonstrate a conductivity increase in comparison with an initial state. The aim of this work was to confirm experimentally the phenomenon of the PbTe resistivity reduction after anodic electrochemical treatment.

## Methods

Monocrystalline (111)-oriented n-PbTe films with 2.3-μm thickness (*d*_init_) were grown on СaF_2_/Si(111) substrates using molecular beam epitaxy in ETH, Zürich [4]. The typical thickness of the calcium fluoride (CaF_2_) buffer insulating layer was 2 to 4 nm. The silicon substrate resistivity was 10^3^ Ω·cm. The measurements of the electrical parameters (resistivity and Hall effect) were carried out in a lateral direction using a four-probe method and a classic Hall method at constant current and constant magnetic field. Magnetic field during the Hall coefficient determination was 0.2 T. The high value of the silicon substrate resistivity and the presence of the calcium fluoride buffer layer allowed us to omit leakage currents to the substrate from consideration. The resistivity of the initial lead telluride films (*ρ*_init_) was (9.6 ± 0.3)·10^−2^ Ω·cm at 300 K.

The one particular feature of the studied PbTe layers, as distinct from our previous work [3], was the standing of the samples at 300 K in the mixed conductivity region, which provided high sensitivity to a possible change of carrier concentration. The PbTe films at low temperatures (15 K) had a p-type conductivity and hole concentration of 1.2·10^17^ cm^−3^. During the temperature increase, a transition to the region of mixed electron–hole conductivity took place, and the conductivity-type inversion effect was observed. The inversion phenomenon is related to the fact that, in lead telluride, the electron mobility exceeds the hole mobility. The studied lead telluride samples at room temperature had an effective n-type conductivity. Hall coefficient value at 300 K was *R*_H init_ = −2.6·10^−6^ m^3^·C^−1^. Since the Hall effect measurements in this case correspond to the region of mixed conductivity, it is not possible to determine the charge carrier concentrations.

**Figure 1:**
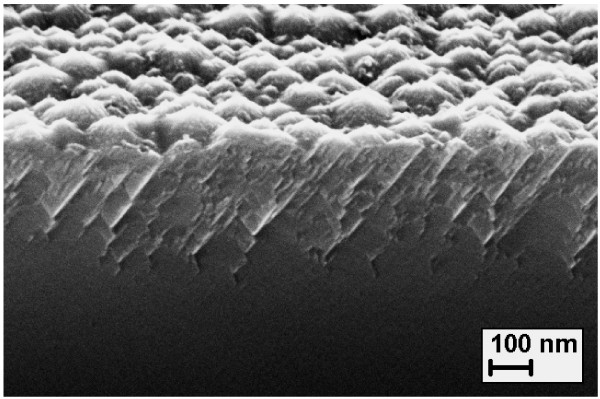
**Typical electron microscopic image of the PbTe anodized sample cross-section.** Scanning electron microscopy image of a cross-section of the PbTe porous layer on CaF_2_/Si(111) substrate fabricated using anodic electrochemical treatment with *j*_а_ = 2 mA·cm^−2^ and *t*_а_ = 10 min (sample tilt is 70°) [6].

The appointed range of the anodizing current density (*j*_а_) during the anodic electrochemical treatment of PbTe films was 2 to 4 mA·cm^−2^. Anodizing time (*t*_а_) was 10 to 20 min. The Norr solution [5] containing 20 g of potassium hydroxide (KOH), 45 ml of distilled water, 35 ml of glycerol, and 20 ml of ethanol was used as an electrolyte. The conditions of the anodic electrochemical treatment for the particular studied samples are summarized in Table 1. The results of the structural and morphological parameter investigations for the porous layers synthesized under these conditions are described in [6,7]. The surficial mesoporous nanostructured layer had a thickness (*d*_por_) up to 200 nm. Due to the etch removal of the material, a decrease of the overall film thickness (*d*) with respect to the initial thickness (*d*_init_) took place during anodic treatment. The typical electron microscopy image of the anodized sample cross-section is shown in Figure 1. The porosity value, as determined with X-ray reflectometry method, was 41% to 52%. The experimental setup, measurement technique, and working formulas for determining the porosity of the samples by X-ray reflectometry are described in full detail in [6,7]. The effective conductivity type of the PbTe films after treatment did not change and remained n-type.

**Table 1:** Pore fabrication conditions for PbTe films

**Sample number**	***j***_**a**_**(mA·cm**^**−2**^**)**	***t***_**a**_**(min)**	***d*(nm)**	***d***_**por**_**(nm)**	**Porosity (%)**
1	2	10	510	180	41
2	2	20	790	160	52
3	4	18	440	140	52

*j*_a_, anodizing current density; *t*_a_, anodizing time; *d*, thickness; *d*_por_, thickness of the porous layer.

## Results and discussion

**Figure 2:**
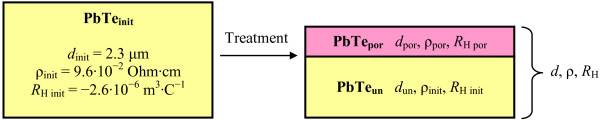
**Parameters for the calculation of the resistivity and Hall coefficient for the double-layered samples.** Shorthand notations for the parameters of the initial PbTe film and the double-layered structure of porous PbTe layer on top of the unmodified PbTe film after anodic electrochemical treatment for the analysis of the resistivity and Hall coefficient change during pore formation. *d*_init_, initial thickness; *ρ*_init_, resistivity of the initial lead telluride films; *R*_H init_, initial layer Hall coefficient; *d*_por_, thickness of the porous layer; *ρ*_por_, resistivity of the porous layer; *R*_H por_, porous layer Hall coefficient; *d*_un_, thickness of the underlying layer.

The results of the measurements of the resistivity (*ρ*) and the Hall coefficient (*R*_H_) for the double-layered samples (Figure 2) after electrochemical treatment were analyzed in the framework of the Petritz’s two-layer model [8], and they are listed in Table 2. During these calculations, the thicknesses of the porous (*d*_por_) and the underlaying monocrystalline PbTe layers (*d*_un_) were considered, and the resistivity value and *R*_H_ for the latter were assumed to be equal to the initial PbTe film values *ρ*_init_ and *R*_H init_. The resistivity of the porous layer was calculated as:

(1) $$\rho_{\text{por}}=\frac{\rho d_{\text{por}}\rho_{\text{init}}}{\rho_{\text{init}}d-\rho d_{\text{un}}}\text{,}$$

where the overall thickness of the double-layered structure is

(2) $$d=d_{\text{por}}+d_{\text{un}}\text{.}$$

**Table 2:** Electrophysical parameters of the porous PbTe samples

**Sample number**	***ρ*(10**^**−2**^ **Ω·cm)**	***ρ***_**por**_**(10**^**−2**^**Ohm·cm)**	***R***_**H**_**(10**^**−6**^ **m**^**3**^**·C**^**−1**^**)**	***R***_**H por**_**(10**^**−6**^ **m**^**3**^**·C**^**−1**^**)**
1	5.7	3.3	−3.7	−2.9
2	7.3	3.8	−3.5	−3.0
3	6.1	3.4	−0.95	−0.2

*ρ*, resistivity; *ρ*_por_, resistivity of the porous layer; *R*_H_, Hall coefficient; *R*_H por_, porous layer Hall coefficient.

The resistivity of the porous layer for all the studied samples decreased to the values of 3 to 4·10^−2^ Ω·cm. Such decrease of the resistivity during the pore formation is an abnormal and uncharacteristic effect for porous semiconductors. Thus, for porous silicon, the ratio *ρ*_por_/*ρ*_init_ lays in the interval of 1.2 to 10^10^[1,2]. In the discussed experiment, *ρ*_por_/*ρ*_init_ value for PbTe was 0.3 to 0.4. In order to explain the obtained results, it is necessary to consider two opposite processes. Firstly, the formation of the pores inevitably results in the increase of the resistivity of the porous material, while, secondly, the change of the metal/chalcogen ratio in behalf of metal can result both in the increase or decrease of resistivity according to p- or n-type conductivity.

For our previously reported results [3], initial PbTe films, due to the bismuth doping, had an extremely high electron concentration (*n* = 5·10^18^ cm^−3^), and the stoichiometry variation during pore formation did not have a significant impact on the *n* value. As a result, the most appropriate approach for the description of the resistivity increase proved to be the effective medium model. In case when PbTe has n-type conductivity with its value close to the range of mixed conductivity near the conductivity-type inversion temperature, the role of the Pb/Te ratio change towards Pb becomes determining due to the strong influence of the carrier concentration.

The values of the Hall coefficient after anodic electrochemical treatment for the double-layered structure were *R*_H_ = −(0.95 − 3.7)·10^−6^ m^3^·C^−1^. The application of the two-layer Petritz’s model allowed us to determine the values of the Hall coefficient for the porous layers [8]:

(3) $$R_{\text{H}}{}_{\text{ por}}=\frac{\rho_{\text{por}}^{2}}{d_{\text{por}}}\left(\frac{R_{\text{H}}d}{\rho^{2}}-\frac{R_{\text{H}}{}_{\text{init}}d_{\text{un}}}{\rho_{\text{init}}^{2}}\right)\text{,}$$

which were amounted to *R*_H por_ = −(0.2 − 3.0)·10^−6^ m^3^·C^−1^. It is known [9] that, for porous semiconductors, the measured Hall coefficient is proportional to the Hall coefficient of the matrix material with a coefficient that depends on the geometry of pores and their arrangement relative to the magnetic and electric field. For the porosity of 50%, this correction factor lies in the range of 1 to 2. For simplicity of the following estimates, we assume that the Hall coefficient of the matrix is equal to *R*_H por_. Theoretical calculations for the range of mixed conductivity [9], which considered the variation of the conductivity and the Hall coefficient, and the decrease of the charge carrier mobility in the porous medium, showed that during the pore formation under the applied anodic treatment conditions the difference in the concentrations of electrons and holes (*n* − *p*)_por_/(*n* − *p*)_init_ increases 5 to 18 times. Therefore, a comprehensive theoretical analysis of the results of measurements of the Hall effect and conductivity confirms the increase in electron concentration in the studied samples at 300 K in the process of pore formation. The increase of the electron concentration and the fact that, in lead telluride, the electron mobility exceeds the hole mobility [10] result ultimately in an increase in the conductivity of the studied porous material.

It is important to note four critical circumstances in this phenomenon. Firstly, the observed effect of the resistivity decrease of the porous PbTe in comparison with monocrystalline material takes place only under specific conditions associated with lead telluride having n-type conductivity with its value near the conductivity-type inversion point. Secondly, the obtained experimental data on the electrical properties of the porous lead telluride prove that this nanostructured material does not show high resistivity values, as is the case, for example, for porous silicon. For porous lead telluride, the phenomenon of a strong depletion of charge carriers, which is pronounced in porous Si, has not been experimentally observed. The latter circumstance can potentially play a major positive role in porous PbTe-based thermoelectric devices fabrication. Thirdly, the decrease of the resistivity in this particular case can result not only from a stoichiometry change during pore formation but also from a variation of the concentration of the electrically active point defects. Fourthly, the experimentally observed absence of an explicit dependence of the resistivity of porous PbTe on the value of the porosity in the range of 41% to 52% confirms the decisive contribution to the conductivity of the processes of the charge carrier concentration change in comparison with the pore scattering processes.

## Conclusions

We have fabricated porous lead telluride layers with 41% to 52% porosity using anodic electrochemical treatment of PbTe/СaF_2_/Si(111) epitaxial structures in a Norr (KOH-based) electrolyte. The resistivity of the porous layers at 300 K abnormally decreased 2.5 to 3 times in comparison with initial state. In order to explain this result, we have proposed a physical model concerning the role of the Pb/Te ratio change towards Pb and the consequent difference in the concentrations of electrons and holes. The obtained results are interesting from the standpoints of the fundamental study of the electrical properties of the porous binary semiconductor materials and of the potential practical applications in electronic and thermoelectric devices.

## Abbreviations

CaF_2_, calcium fluoride; KOH, potassium hydroxide; PbTe, lead telluride.

## Competing interests

The authors declare that they have no competing interests.

## Authors’ contributions

SPZ designed and carried out the experimental work, conducted basic characterizations of the samples, analyzed all the data, and wrote the manuscript. ESG and FOS performed the microscopic observations and analysis, and obtained the electrical parameters of the samples. All authors read and approved the final manuscript.

## Authors’ information

SPZ is a professor at the Microelectronics Department, Yaroslavl State University. ESG is a principal engineer at the Microelectronics Department, Yaroslavl State University and a research associate at the Yaroslavl Branch of the Institute of Physics and Technology of Russian Academy of Sciences. FOS is a student at the Microelectronics Department, Yaroslavl State University.
